# Breaking the cycle of the COVID-19 transmission: A challenge for Nigeria

**DOI:** 10.7189/jogh.10.020309

**Published:** 2020-12

**Authors:** Bashiru Garba, Zunita Zakaria, Mohammed Danlami Salihu, Faruku Bande, Bashir Saidu, Jamilu Abubakar Bala

**Affiliations:** 1 Department of Veterinary Pathology and Microbiology, Faculty of Veterinary Medicine, Universiti Putra Malaysia, Serdang, Selangor, Malaysia; 2 Institute of Bioscience, Universiti Putra Malaysia, Serdang, Selangor, Malaysia; 3 Department of Veterinary Public Health & Preventive Medicine, Faculty of Veterinary Medicine, Usmanu Danfodiyo University Sokoto, Sokoto State, Nigeria; 4 Department of Veterinary Microbiology, Faculty of Veterinary Medicine, Bayero University Kano, Kano State, Nigeria; 5 Department of Veterinary Physiology & Biochemistry, Faculty of Veterinary Medicine, Usmanu Danfodiyo University Sokoto, Sokoto State, Nigeria; 6 Department of Medical Laboratory Science, Faculty of Allied Health Sciences, Bayero University Kano, Kano State, Nigeria

COVID-19 has already spread to almost every country in the world, including the arctic. The impact on human health has been severe, with an increasing number of fatalities. With the spread comes economic hardship due to the preventive strategies adopted. Movement restrictions imposed in Nigeria as a result of the outbreak have generated controversies among the poor masses that depend on daily hustles to fend for themselves and their families. Nigeria being a very populous country with a high number of low-income earners who depend on their daily efforts to get food for their families, control measures like the movement restrictions and closure of business premises would have a devastating impact on them. Although the government has responded with some palliative measures, it is evident that these interventions may not be sufficient, mainly due to potential malpractices that will end up denying many supposed beneficiaries.

According to the WHO COVID-19, situation report-133, as of 1 June 2020, a total of 6 057 853 confirmed cases of COVID-19, including 371 166 deaths has been reported globally [1]. Many African countries, including Nigeria, are seriously battling with the disease amidst lack of resources, lack of technical expertise, high population density and lack of proper awareness. The first case of the novel coronavirus disease (COVID-19) in Nigeria was confirmed on the 27 of February 2020. However, as of 2 June 2020, the Nigerian Centre has reported a total of 10 162 confirmed cases with 287 deaths across the 35 states of the federation and the federal capital territory (**Figure 1**). Ironically, only 63 882 tests have been conducted nationwide for a population of over 200 million. This is grossly inadequate considering that the country has been classified to already have community transmission of the virus infection; however, the lack of enough testing facility and properly coordinated response may have contributed to this outcome. The cases involved 35 States with Lagos, Kano, and Abuja leading with the majority of cases (4943, 954, and 660, respectively). The initial trend of infection indicates that most of the patients had travel history from countries that had earlier recorded the outbreak, however recently the WHO has confirmed that community transmission has ensued [3].

**Figure 1:**
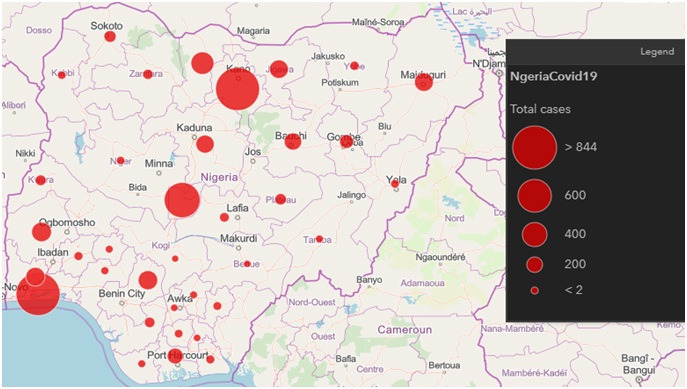
COVID-19 dot-map indicating the number of confirmed cases in Nigeria, 2 June 2020 [2].

As the disease continues to ravage most parts of Nigeria, there is growing concern that the country is facing serious health and humanitarian crises. This can be attributed to the inherent poor health care delivery services, poverty, as well as the large number of individuals living in close proximities, especially in rural areas and urban slums [4]. The Nigeria Centre for Disease Control is the country’s national public health institute, tasked with the mandate to lead the preparedness, detection and response to infectious disease outbreaks and public health emergencies (https://ncdc.gov.ng/ncdc). With support from the Federal Government of Nigeria (FGN), the institute is working assiduously in handling the COVID-19 pandemic. This involves rigorous detection strategies by testing, isolating and tracing of in-contact individuals. It also carries public enlightenment campaigns and mobilisation of citizens to observe the standard prevention and control measures in response to the outbreak. The NCDC has made considerable progress by strengthening its laboratory diagnostic capacities from an initial six NCDC molecular laboratory network located mainly in the Southwestern states and the Federal Capital Territory, to 29 (31-05-2020) distributed across the six geopolitical zones of the country, with additional two (Kwara and Gombe States) at the verge of completion.

Although no vaccine is currently available for the disease, patients are mainly treated symptomatically and given supportive therapy; and so far the approach seems to be working having recorded 3007 recovered and discharged patients (29.6%) with 287 (2.8%) deaths as of 2 June 2020, which is relatively fair compared to many other countries including the United States, Brazil and United Kingdom [1]. Like most countries battling the disease, Nigeria has resorted to using a combination of containment and mitigation strategies in order to forestall further spread of the disease. These strategies aimed at slowing down surges of patients and preventing the over-stretching of the available health care facilities include; initial screening of individuals showing signs of illness synonymous with COVID-19 with or without a history of travel to or from high-risk countries, total suspension of all local and international travels, contact tracing and mandatory self-isolation for 14 days for individuals with history of travel or signs of illness, advocacy for personal hygiene and general cleanliness as well as the unpopular social distancing and/or banning of large gatherings or religious congregations.

## MAJOR CHALLENGES DURING THE COVID-19 OUTBREAK IN NIGERIA

In the last two decades, emerging and re-emerging infectious disease are occurring in Nigeria in epidemic proportions and at a devastating public health scale [5]. Popular among the most recent epidemics are Ebola, Yellow fever, Monkey pox, and sporadic episodes of Lassa fever [6]. This is including the dreaded Malaria that has become endemic. The lessons drawn from these outbreaks are believed to have helped in managing the present pandemic caused by the SARS-CoV-2 virus. Recall that during the Ebola outbreak, considerable political and public health resources were rapidly deployed, resulting in the timely and effective containment of the disease [5].

At present, Nigeria have progressed to the dreaded community transmission stage as far as COVID-19 is concerned [7]. This evidently manifested following the confirmation of COVID-19 cases from patients with no history of travel to neither an infected country/state nor contact with known confirmed COVID-19 case; thus the dynamics of transmission imply that social interactions, potential exposures in health care settings and even family events have been the major triggers to several unconfirmed cases. Given the complex nature of the country, large size population, poor health care system manned by inadequate diagnostics and isolation centers, as well as corruption; fighting this public health crisis is therefore fraught with numerous challenges including; poor preparedness and response plan. The socio-cultural and religious beliefs, porous nature of international and interstate borders as well as lack of synergy between federal and state governments may in part, affect policies directly intended to reduce the spread of the virus. No doubts that measures related to self-isolation/quarantine, social distancing, and movement control would be key in this directions, nonetheless the measures would be greeted with a lot of resistance and criticisms from the populace due to poor public health information dissemination and economic challenges.

**Figure:**
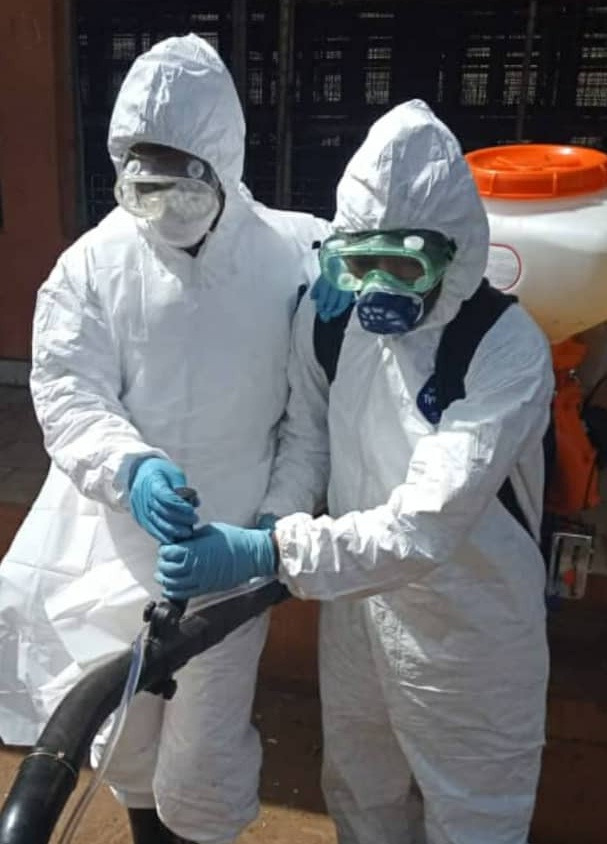
Photo: Team of infectious disease unit staff, Department of Veterinary Services, Sokoto State, Nigeria, disinfecting the live bird market premises and informing the public on the importance of hygiene in curbing the spread of COVID-19 and other infectious pathogens (from the author’s collection, used with permission).

## PROSPECT AND FUTURE DIRECTIONS

In the face of an impending economic crisis and recession occasioned by the halt in economic activities due to the pandemic, many countries including Nigeria are already considering relaxing the movement control orders with a view to reviving the economy and well-being of its people. Unfortunately, cases are still on the increase. Nonetheless, because of the potential civil unrest that may trail the continued lockdown caused by the inability of the government to adequately provide the needs of the majority of the population, the Nigerian government may be right to equally consider relaxing the movement restriction, but with some precautions. Some of the measures that may assist in the fight against this scourge and recovery may include; relaxation of the lockdown and the enforcement of social distancing. With social distancing rules in place and strictly adhered to, complimenting it with intensive testing, isolation and contact tracing of new infections will go a long way in containing the spread of the virus. Nigeria can equally employ the use of non-pharmaceutical strategies for disease containment and mitigation such as the use of digital technology in contact-tracing which has proven to be a success in some countries like, Singapore [8]. Similarly, the use of alternative non-laboratory-based rapid easy-to-use devices such as the point-of-care lateral flow immunoassay kit, and the field-effect transistor (FET)-based bio sensing device should be explored in order to scale up the country’s testing capacity, identify true infection rate and address the growing number of COVID-19 cases [9,10]. In the same vein, there is the need to improve on effective risk communication to enhance public education on the mode of transmission of the disease as well as the importance of personal hygiene, physical distancing and other modes of preventions. The need for political leaders and public health experts to furnish the public with clear, honest and verified information is considered one of the most effective strategies for prevention against the disease [11]. Health communications can enhance how communities respond to the uncertainties and fears associated with the COVID-19 and encourage compliance to the necessary behavioural changes (use of facial masks, physical distancing, and frequent hand washing) especially among vulnerable groups with co-morbidity or underlining courses. These steps are critical, as we are likely to live with the disease for a long period of time.

Although the technical knowledge of the virus and its pathogenesis is still not fully understood. Hence, dealing with a pandemic of this nature will require a strong and comprehensive skill. Critical capacity building and training of health workers is an essential component of public health emergencies. Therefore, the government must ensure a robust and standardised training on critical-care medicine so as to improve the knowledge and capacity of the front line health care service providers so that they will be better prepared to deal with the current pandemic. On the other hand, the halt on movement and economic activities may be seen as a good strategy to stop the spread of coronavirus, but for the impoverished masses in Nigeria, it is simply not a realistic option unless the government is ready to do the needful through the provisions of palliatives and cushioning supports to individuals and other critical sectors of the economy such as agriculture and essential commodities.

In conclusion, Nigeria, like other developing economies is indeed facing tough times as a result of the COVID-19 pandemic, and it is likely that transmission of the disease will continue amidst other growing challenges. Invariably, tackling these challenges would require a holistic approach involving non-partisan and well-coordinated national response from the government end. The government should intensify its efforts to enlighten its citizens on the importance of personal hygiene, undertake mass health education at the grass root level, and encourage rapid surveillance system rather than the selective testing currently being practiced. While, private organizations and individuals in the society are enjoined to take care of their personal health and their personal wealth, they are to also support the government’s efforts as it makes painful adjustments to cope with the realities and overcome the challenges associated with the present pandemic; hopefully we have learnt a lesson.
